# 2021 Editors’ Highlights

**DOI:** 10.1038/s42004-021-00606-y

**Published:** 2021-12-14

## Abstract

The Editors and Editorial Board of *Communications Chemistry* are pleased to launch a 2021 Editors’ Highlights collection featuring some of their favourite Articles published in the journal this year. Here we highlight each Article and outline why it was selected.

Chief Editor Dr Victoria Richards highlights ‘Atomic resolution tracking of nerve-agent simulant decomposition and host metal–organic framework*’* by Sanjit Ghose, Anatoly Frenkel and colleagues (10.1038/s42004-020-00439-1). “The team elegantly combine X-ray total scattering measurements, pair distribution function analysis and theoretical calculations to quantitatively track the interactions between a Zr-based metal–organic framework host and a nerve-agent simulant guest with atomic resolution. The insight gained could prove valuable towards the goal of using framework materials to capture and decompose chemical warfare agents.”

Editorial Board Member Dr François-Xavier Coudert highlights ‘Local structure and distortions of mixed methane-carbon dioxide hydrates’ by Claudia Rawn and colleagues (10.1038/s42004-020-00441-7). “The team uses a combination of neutron scattering experiments, reverse Monte Carlo fitting, and molecular dynamics simulations to investigate the atomic structure of mixed methane-carbon dioxide hydrates. This is a captivating fundamental study, with a complex behaviour in a relatively simple system, in an area of huge significance in the context of global warming.”

Editorial Board Member Dr Kristin Wustholz highlights ‘Rotaxane rings promote oblique packing and extended lifetimes in DNA-templated molecular dye aggregates*’* by Ryan Pensack and colleagues (10.1038/s42004-021-00456-8). “The team report a controlled aggregation strategy based on DNA templating of squaraine dyes functionalized with rotaxane rings, which yields an oblique aggregate with a prolonged excited-state lifetime and reduced structural heterogeneity. This may offer a general strategy for optimizing excitonic materials for solar energy conversion and nanoscale computing applications.”

Editorial Board Member Dr Satoshi Honda highlights ‘Supramolecular construction of a cyclobutane ring system with four different substituents in the solid state’ by Ryan Groeneman, Leonard MacGillivray and colleagues (10.1038/s42004-021-00493-3). “The team exploit principles of crystal engineering and supramolecular chemistry to construct cyclobutanes with four different substituents via [2 + 2] cross-photoreaction of functionalized alkenes. This approach could be useful for constructing chemically and biologically important building blocks.”

Editorial Board Member Dr Kui Yu highlights ‘Functionalized Au_15_ nanoclusters as luminescent probes for protein carbonylation detection’ by Vlasta Bonačić-Koutecký, Rodolphe Antoine and colleagues (10.1038/s42004-021-00497-z). “The team report the design and synthesis of gold clusters with glutathione (SG) ligands, Au_15_SG_13_. In order to monitor protein carbonylation, each cluster possesses a thiolated aminooxy moiety. Such functionalization, which imparts protein carbonyl-binding properties, enables the use of luminescent clusters as contrast agents in life science applications.”

Editorial Board Member Dr David Nelson highlights ‘Air-stable 18-electron adducts of Schrock catalysts with tuned stability constants for spontaneous release of the active species’ by Henrik Gulyás, Imre Pápai and colleagues (10.1038/s42004-021-00503-4). “The team design a series of new user-friendly pre-catalysts that release Shrock-type alkene metathesis catalysts in situ by dissociation of a chelating bidentate *N*,*N*-ligand. The careful selection of a ligand with a finite association constant means that a Lewis acid additive is not required to release the active catalyst, and that the equilibrium between pre-catalyst and active catalyst can be manipulated by changing the reaction concentration. This could allow a range of non-specialist researchers to handle and use these highly active metathesis catalysts in synthetic organic and polymer chemistry, without recourse to using a glovebox.”

Senior Editor Dr Andrew Bissette highlights ‘Reaction-diffusion hydrogels from urease enzyme particles for patterned coatings*’* by Annette Taylor, John Pojman Sr. and colleagues (10.1038/s42004-021-00538-7). “The team report the use of enzyme-generated pH gradients to promote the rapid growth of micrometer-thick hydrogel layers around urease-rich magnetic particles, which can produce layered patterns that are dependent on the spatial configuration of the particles. This may offer a general approach for the controlled synthesis of patterned and layered hydrogels with spatial and temporal control.”

Editorial Board Member Dr Jennifer Bridwell-Rabb highlights ‘Exo-selective intermolecular Diels-Alder reaction by PyrI4 and AbnU on non-natural substrates’ by Anthony Addlagatta and colleagues (10.1038/s42004-021-00552-9). “The team uses enzyme assays and structural biology to show that two naturally occurring enzymes can be used to catalyze stereospecific Diels-Alder reactions on non-natural substrates. This work reveals the utility of the existing so-called Diels-Alderases that are found in nature and highlights how they could be used as tools for efficient and stereoselective industrial production of complex molecules.”

Editorial Board Member Dr Jun Lu highlights ‘The electric double layer effect and its strong suppression at Li^+^ solid electrolyte/hydrogenated diamond interfaces’ by Takashi Tsuchiya and colleagues (10.1038/s42004-021-00554-7). “The electric-double-layer (EDL) effect at hydrogenated diamond-based transistors is investigated on the basis of electric conduction characteristics. The correlation of thermal activation behavior and EDL-induced electronic carrier accumulation to ionic conductivity is revealed by advanced in situ hard X-ray photoelectron spectroscopy. The present technique is potentially very useful for revealing EDL behavior in the vicinity of solid/solid electrolyte interfaces and for clarifying the effect of the interface characteristics on the total performance of solid-state ionic devices.”

Editorial Board Member Professor Yanli Zhao highlights ‘Solvophobicity-directed assembly of microporous molecular crystals’ by Hiroshi Yamagishi and colleagues (10.1038/s42004-021-00561-8). “Supported by detailed computational studies and crystal structures, the team assembles designed aromatic molecules into isostructural porous polymorphs. They find that solvophobicity plays a key role in directing the superstructure formations. This work sheds light on developing porous organic crystalline materials by exploiting solvophobic effects.”

Senior Editor Dr Teresa Ortner highlights ‘Phase transition mechanism and bandgap engineering of Sb_2_S_3_ at gigapascal pressures’ by Qingyang Hu and colleagues (10.1038/s42004-021-00565-4). “The team apply external stress to destabilize antimonite and study its structural evolution using spectroscopic and computational methods. This insight could inform future efforts to tune this abundant, non-toxic material to make it a competitive thermoelectric or solar absorber material.”

Editorial Board Member Dr María Escudero-Escribano highlights ‘Selective electrochemical oxidative coupling of methane mediated by Sr_2_Fe_1.5_Mo_0.5_O_6-δ_ and its chemical stability’ by Fernando Garzón and colleagues (10.1038/s42004-021-00568-1). “The team investigates the performance of Sr_2_Fe_1.5_Mo_0.5_O_6-δ_ (SFMO) for electrochemical methane activation and oxidation to valuable products. Interestingly, online mass spectrometry measurements show the activation of methane at low overpotential on SFMO. The insight gained highlights the importance of electrochemical methods in controlling methane activation and oxidation as well as the need to discover active, selective and stable electrocatalyst materials.”

In addition to the many notable primary research Articles published in the journal this year, we are pleased to have published a number of timely and informative Review articles as well as valuable opinion pieces from the community in the form of Comment articles. It is our pleasure to highlight these contributions in our collection alongside the above described research Articles.

